# Overview of approaches to estimate real-world disease progression in lung cancer

**DOI:** 10.1093/jncics/pkad074

**Published:** 2023-09-21

**Authors:** Rossybelle Amorrortu, Melany Garcia, Yayi Zhao, Issam El Naqa, Yoganand Balagurunathan, Dung-Tsa Chen, Thanh Thieu, Matthew B Schabath, Dana E Rollison

**Affiliations:** Department of Cancer Epidemiology, Moffitt Cancer Center, Tampa, FL, USA; Department of Cancer Epidemiology, Moffitt Cancer Center, Tampa, FL, USA; Department of Cancer Epidemiology, Moffitt Cancer Center, Tampa, FL, USA; Department of Machine Learning, Moffitt Cancer Center, Tampa, FL, USA; Department of Machine Learning, Moffitt Cancer Center, Tampa, FL, USA; Department of Biostatistics and Bionformatics, Moffitt Cancer Center, Tampa, FL, USA; Department of Machine Learning, Moffitt Cancer Center, Tampa, FL, USA; Department of Cancer Epidemiology, Moffitt Cancer Center, Tampa, FL, USA; Department of Cancer Epidemiology, Moffitt Cancer Center, Tampa, FL, USA

## Abstract

**Background:**

Randomized clinical trials of novel treatments for solid tumors normally measure disease progression using the Response Evaluation Criteria in Solid Tumors. However, novel, scalable approaches to estimate disease progression using real-world data are needed to advance cancer outcomes research. The purpose of this narrative review is to summarize examples from the existing literature on approaches to estimate real-world disease progression and their relative strengths and limitations, using lung cancer as a case study.

**Methods:**

A narrative literature review was conducted in PubMed to identify articles that used approaches to estimate real-world disease progression in lung cancer patients. Data abstracted included data source, approach used to estimate real-world progression, and comparison to a selected gold standard (if applicable).

**Results:**

A total of 40 articles were identified from 2008 to 2022. Five approaches to estimate real-world disease progression were identified including manual abstraction of medical records, natural language processing of clinical notes and/or radiology reports, treatment-based algorithms, changes in tumor volume, and delta radiomics–based approaches. The accuracy of these progression approaches were assessed using different methods, including correlations between real-world endpoints and overall survival for manual abstraction (Spearman rank ρ = 0.61-0.84) and area under the curve for natural language processing approaches (area under the curve = 0.86-0.96).

**Conclusions:**

Real-world disease progression has been measured in several observational studies of lung cancer. However, comparing the accuracy of methods across studies is challenging, in part, because of the lack of a gold standard and the different methods used to evaluate accuracy. Concerted efforts are needed to define a gold standard and quality metrics for real-world data.

The Food and Drug Administration defines real-world data as those that “relate to patient health status and/or delivery of health care routinely collected from a variety of sources, including the electronic health record (EHR), claims/billing, disease registries, patient-reported data, and other health data such as those obtained from mobile devices” (1). The potential to leverage real-world data to accelerate clinical trials with historical control groups (2) and advance biomedical research through considerably large datasets comprised of diverse patient populations is tremendous; however, challenges in data quality, completeness, and lack of standardization in clinical documentation are important impediments to the realization of this potential (3). Unlike other chronic diseases (eg, cardiovascular disease and diabetes) where disease status and progression can be accurately measured through discrete measurements or laboratory values, the challenges concurrent with real-world data are particularly pronounced in oncology given that the severity of cancer is measured through a variety of means. For instance, in most solid tumors, radiological observation (4-6) remains the primary response assessment, whereas other targeted biomarkers, including circulating tumor markers, are also used but are often summarized in unstructured clinical notes (7). Even when the information can be successfully extracted, there is little consensus as to how cancer progression should be defined, especially when considering heterogeneity across cancer sites.

Within randomized clinical trials (RCTs) of novel treatments for solid tumors, disease progression is typically measured using a widely adopted and validated gold standard called the Response Evaluation Criteria in Solid Tumors (RECIST) (4). Patient assessments (eg, imaging, laboratory tests) are precisely timed relative to treatment administration, and cancer status is defined by using either standard RECIST criteria (8) or modified versions of the RECIST criteria (9). Information on disease progression is then manually abstracted from the EHR into case reports forms, with these highly curated data being used for statistical analysis (8). Outside of RCTs, however, patient assessments are not precisely timed, uniformly documented, or manually curated at scale, posing challenges to obtain information on disease progression using real-world data. Efforts to create standardized coding frameworks for real-world data–derived cancer outcomes have prompted the development of composite progression metrics such as Flatiron Health’s “real-world disease progression” (10,11), minimal common data elements (mCODE’s ) disease status (12), and pathology, radiology/imaging, signs/symptoms, medical oncology’s (PRISSMM) cancer progression (13,14). Although these coding frameworks can be used to guide manual abstraction of real-world data from the EHR to acquire outcome metrics (15,16) such as cancer progression, manual abstraction is highly resource intensive and difficult to scale across a wide range of clinical settings and patient populations. Hence, novel approaches using informatics-based approaches to estimate disease progression at scale may advance the use of real-world data for the acceleration of cancer research. The purpose of this narrative review is to summarize current methods to estimate cancer disease progression using real-world data, including text-based review of clinical notes or EHR (eg, manual abstraction of medical records, natural language processing [NLP] to identify disease progression in clinical notes and/or radiology reports), algorithms of changes in treatment, changes in tumor volume, and delta radiomics–based approaches, highlighting illustrative examples in lung cancer.

## Methods

To identify research articles that used novel real-world approaches to estimate disease progression, a narrative literature review (17,18) was conducted using peer-reviewed publications indexed in the PubMed database, in contrast to systematic reviews that identify all existing relevant research articles following comprehensive guidelines (17,18). The current narrative review sought to provide a broad overview of prior methods used to define cancer progression using real-world data using lung cancer case examples identified in the literature, discuss challenges and limitations of each approach, and propose future directions for the advancement of persistent measurement challenges.

### Narrative review strategy

A narrative review of the existing literature was conducted in the PubMed database. Authors (RA, MG, YZ, DR) identified potentially relevant key search terms, and using an iterative approach, 2 reviewers (RA and MG) queried on various combinations of search terms, seeking those that yielded the most relevant articles. After both reviewers tested multiple search combinations and investigated the database results for unique articles, the following combinations of terms were finalized for use in the narrative review: (“Real-world data” AND “progression” AND “lung cancer”), (“radiomics” AND “response” AND “lung cancer”), (“image segmentation” AND “response” AND “lung cancer”), and (“real-world treatment patterns” AND “progression” AND “lung cancer”). Searches were performed for each of the identified combinations separately and exported into a comprehensive Endnote 20 library. After excluding duplicated articles, 971 unique articles were identified.

### Article selection criteria and screening process

Articles were eligible for inclusion in our narrative review if they were 1) original research articles, 2) published in the English language between January 2008 and December 2022, and 3) either specifically tested or developed real-world methods to estimate disease progression in lung cancer patients or used real-world endpoints in the context of a specific research question related to lung cancer. Articles were excluded if they 1) focused solely on non-progression metrics (eg, disease incidence), 2) examined real-world progression for other cancers, 3) did not provide a definition of how real-world disease progression was defined in their study, 4) extracted radiomics features to predict disease progression, 5) were published before 2008 or after 2022, 6) were not written in English, or 7) were not original research articles (Supplementary Figure 1, available online).

After articles were identified from the search strategy described above, the following screening process was performed. First, abstracts from the 971 unique articles were screened by 2 reviewers (RA and MG) for potential relevance. Full-text review was subsequently conducted for 141 unique articles, and the 2 reviewers discussed each article to develop a consensus for eligibility for inclusion in the narrative review. The reference lists of included articles were also examined by the reviewers to identify additional relevant studies (see Supplementary Figure 1, available online, for further detail).

### Data abstraction framework for identifying relevant articles

A data abstraction framework was constructed by reviewers a priori to facilitate the abstraction data on 1) source of clinical data used, 2) description of the method used to estimate real-world progression, 3) whether the real-world endpoints were compared with a selected gold standard and the results of that comparison, 4) the definition of the selected gold standard (if applicable), and 5) a consensus statement by both reviewers indicating whether the article should be included or excluded from the narrative review. This data abstraction framework was used by both reviewers (RA and MG), and all data extraction components were manually inputted in a Microsoft Excel spreadsheet.

## Results

A total of 40 articles were included in the review, incorporating 5 approaches to estimate real-world progression in lung cancer including 1) manual abstraction of medical records, 2) NLP to identify disease progression in clinical notes and/or radiology reports, 3) algorithms of changes in treatment, 4) changes in tumor volume, and 5) delta radiomics–based approaches.

### Text-based review of clinical notes or EHR

#### Manual abstraction of medical records

Table 1 lists the studies that conducted manual review and abstraction of medical records to estimate disease progression in lung cancer patients. For a majority of these articles, the focus was less on the method of real-world progression assessment or estimation and more so on analyses that used real-world progression as an endpoint (19-32). Definitions of disease progression varied across the studies identified, including the types of information from the medical record used during the data abstraction process. For example, some studies (10,11,19–21,33–36) based disease progression on clinical notes alone, whereas others based disease progression on combinations of radiology reports, pathology reports, and/or clinical notes (22,37–40). Specific details as to how disease progression was classified using these notes were described vaguely for some studies, limiting the reproducibility of these approaches by others (38,39,43,44). Some studies attempted to examine the validity of the manually abstracted cancer progression outcomes by examining their correlation with overall survival (10,11,34,43,45,46). The correlation between overall survival and real-world progression was lowest (Spearman rank ρ = 0.61) in a study that measured time to progression defined as the time between initiation of the study treatment and the documented date of progression in the clinic note (13), while the highest correlation (Spearman rank ρ = 0.84) was reported by a study that measured real-world progression-free survival (PFS) as clinician-documented tumor growth or worsening of the cancer per the patient record (43). Despite the dissimilarities in manual abstraction approaches, manual abstraction was most often used to define the gold standard datasets for the training and/or validation of more automated algorithms to approximate cancer disease progression in the articles described below.

**Table 1.** Example of studies using manual abstraction approaches to estimate disease progression in lung cancer patients

Author	Cancer type	Institution	Definition of disease progression	Comparison to a selected gold standard	Results
Fox et al. (2008) (36)	NSCLC	Henry Ford Health System	Change in chemotherapy regimen and documented tumor growth on radiology report. Patients with bronchioloalveolar carcinoma who had a documented chemotherapy change were required to have documented worsening pain or shortness of breath in the clinical note, because this carcinoma does not have radiologic presentation.	NA	NA
Lunacsek et al. (2016) (33)	NSCLC	International Oncology Network	Real-world PFS defined as the time from index date to progression determined from clinical notes as mention of disease progression or mixed response, initiation of hospice, or death.	NA	NA
Coutinho et al. (2019) (19)	Small cell lung cancer	International Oncology Network Clinical Oncology Database	Progression was defined within standardized fields in the progress notes of patient medical charts and extracted during the review process; no additional details on the abstraction process given. Real-world PFS, defined as the time from initiation of cancer therapy until whichever date came first: 1) date of identified progression, 2) date of death, 3) loss to follow-up, or 4) end of study.	NA	NA
Davis et al. (2018) (20)	NSCLC	Canada centers	Real-world PFS, defined as the time from crizotinib until 1) physician documented progression from patient medical records or 2) death, whichever came first.	NA	NA
Skinner et al. (2018) (38)	NSCLC	Vector Oncology	Progression determined from pathology reports, radiology scans, lab values, or clinical notes.	NA	NA
Vilhonen et al. (2019) (37)	Lung cancer	Turku University Hospital	Real-world PFS, defined as a mention of progression in patient records (ie, radiological, pathological, or clinical). If progression was unclear, physician retrospectively reviewed radiological images using the RECIST criteria.	NA	NA
Li et al. (2020) (35)	Lung cancer	Mayo Clinic	RECIST-inspired response from clinical notes was categorized into 4 response levels: Complete response: disappearance of all target lesions; any pathologic lymph nodes must have reduction in the short axis to 10 mm. Partial response: 30% or more decrease in the sum of diameters of target lesions. Progressive disease: 20% or more increase in the sum of diameters of target lesions and an absolute increase of at least 5 mm. The appearance of 1 or more new lesions is also considered progression. Stable disease: neither sufficient shrinkage to qualify for partial response nor sufficient increase to qualify for progressive disease.	NA	Created gold standard dataset of 609 cases that capture all relevant information to define a RECIST-inspired response from clinical notes. These manually abstracted real-world endpoints are intended to be used as a training dataset for the development of future NLP models.
Ma et al. (2021) (10)	NSCLC	Flatiron Health	Progression was defined as the clinician’s assessment of change in cancer burden in an individual line of therapy. First, imaging time points were identified within a line of therapy, which were anchored by the imaging report date. Clinical notes corresponding to the time point were reviewed to document changes in cancer burden and categorized as real-world complete response, real-world partial response, real-world stable disease, real-world progressive disease, real-world pseudoprogression, indeterminate response, not documented.	NA^a^	At 3-months, patients responding showed a statistically signficant lower risk of death and progression in real-world overall survival (HR = 0.68, 95% CI = 0.58 to 0.73) and real-world PFS (HR = 0.65, 95% CI = 0.59 to 0.72). A strong correlation was observed between real-world response rates (from 12 real-world cohorts) and overall response rates in randomized trials (Spearman *q* = 0.99).
Arbour et al. (2021) (44)	Lung cancer	Memorial Sloan Kettering	Manual review of radiology reports performed by 2 trained abstractors; no additional details given.	Radiologist determined RECIST best overall response, occurrence of progression	Manual review correctly predicted RECIST best overall response in 92% of patients, progression in 90% of patients, and exact date of progression in 79% of patients.
Kehl et al. (2021) (45)	NSCLC	Memorial Sloan Kettering, Dana Farber Cancer Center, Vanderbilt University	Four pathology, radiology/imaging, signs/symptoms, medical oncologist assessment, and biomarkers–derived PFS outcomes: 1) PFS imaging, defined as the time to worsening/progression as documented in imaging report or death; 2) PFS medical oncologist, defined as the time to worsening/progression as documented in oncologist assessment or death; 3) PFS imaging or medical oncologist, defined as worsening and/or progression as documented in imaging report or oncologist assessment; 4) PFS imaging and medical oncologist, defined as worsening/progression as documented in both imaging report and oncologist assessment.	NA^a^	Correlation between overall survival and real-world PFS, was ρ = 0.63 for PFS imaging; ρ = 0.71 for PFS-medical oncologist; ρ = 0.61 for PFS imaging or medical oncologist; and ρ = 0.76 for PFS imaging or medical oncologist.
Danesi et al. (2022) (21)	NSCLC	IRCCS Instituto Romagnolo, Italy	Real-world PFS, defined as the time from first dose of 1 L treatment to clinician-documented clinical disease progression, initiation of new line of therapy, or death from any cause, whichever came first.	NA	NA
Goto et al. (2022) (22)	NSCLC	Japan hospitals and medical centers	Real-world PFS, defined as time from initiation of pembrolizumab to clinically or radiologically documented disease progression or death, whichever came first. Real-world tumor response rate, defined as the proportion of patients with a radiologically or clinician-documented tumor response classified as complete response or partial response.	NA	NA
Izano et al. (2022) (42)	Lung cancer	Synapse Learning Health Network	Imaging time points were defined and all scans in that time frame were evaluated as a scan bundle. At each time point response was determined using 2 methods: 1) response as documented in the clinical notes using a set of key terms and 2) response as documented in the radiologists’ reports. Response at each time point was categorized as complete or partial response, stable disease, progressive disease, or not evaluable.	Radiologist’s reassessment of archived images using RECIST v1.1 criteria as adapted by study team for use with real-world data.	NA
Van Dao et al. (2022) (23)	NSCLC	Vietnam cancer hospitals	Real-world PFS, defined as the time between start of treatment to date of disease progression or death documented in patient medical charts (eg, pathology reports, imaging reports, clinical notes), whichever date came first.	NA	NA
Velcheti et al. (2022) (24)	NSCLC	Flatiron Health	Real-world PFS, defined as the first episode of tumor growth or worsening of disease defined by the treating physician and manually abstracted from the EHR. Real-world tumor response, measured by changes in NSCLC tumor burden as described in the radiology reports and classified as complete response, partial response, stable disease, progressive disease, and other (pseudoprogression, indeterminate, not documented).	NA	NA
Multiple authors (11,25–32,34,37,39–41,46)^b^	NSCLC	Flatiron Health	Real-world PFS, defined as the time from initiation of cancer therapy to disease progression, death, or loss-to follow-up as documented by the treating physician in the EHR (eg, clinical notes, pathology or radiology reports). Time to progression defined as the time between initiation of systemic therapy and date of progression, excluding death as documented by the treating physician in the EHR (eg, clinical notes, pathology or radiology reports)	NA	The correlations between real-world PFS and time to progression with overall survival were positive for most FH studies. (10,11,31,34,43,45,46)^c^ NA

a Study did not use a gold standard; however, they looked at correlations with other cancer endpoints and the manually abstracted progression outcome to “validate” the data. CI = confidence interval; EHR = electronic health record; HR = hazard ratio; IRCCS = Instituto di Ricovero e Cura a Carattere Scientifico;  NA = not applicable; NSCLC = non–small cell lung cancer; NLP= natural language processing; PFS = progression-free survival; RECIST= Response Evaluation Criteria in Solid Tumors.

b The following FH studies were combined due to overlap in real-world endpoints and methods: Griffith, Tucker (2019); Griffith, Miksad (2019); Khozin (2019); Vilhonen (2019); Jahanzeb (2020); Stinchcombe (2020); Dal Maso (2021); Jahanzeb (2021); Stenehjem (2021); Waterhouse (2021); Ge (2022); Ton (2022); Torres (2022); Wu (2022).

c Griffith, Tucker (2019); Griffith, Miksad (2019); Stewart (2019); Khozin (2019); Ma, Bellomo (2021); Kehl, Reily (2021); Torres (2022).

#### NLP to identify disease progression in clinical notes and/or radiology reports

Table 2 lists the studies that employed NLP to extract information from the free text notes of the EHR. Examples of note types used in these NLP methods included radiology reports containing information on changes in tumor size or progression and physician notes containing comments regarding cancer disease progression or recurrence. The studies that used deep learning algorithms reported high levels of accuracy as compared with their respective gold standard (13,44,47). For example, one study that used deep learning models trained on radiology reports reported area under the receiver operator curves of more than 0.9 for worsening and/or progression or improving cancer outcomes (13). A second study by the same authors reported slightly lower area under the receiver operator curves for deep learning models trained on clinical oncology notes, which ranged from 0.86 to 0.90 (47). Two studies by Kehl et al. (13,47) used manually abstracted data following the PRISSMM framework as a gold standard to train the deep learning models, whereas a study by Arbour et al. (44) used RECIST reads performed by a radiologist as the selected gold standard. Despite the differences in the gold standards used across these studies, there was limited variation observed in model accuracy; however, it is unknown how these models would perform head-to-head using a common gold standard for comparison.

**Table 2.** Example of studies using natural language processing approaches to estimate disease progression in lung cancer patients^a^

Author	Cancer type	Institution	Approach to estimate progression	Comparison to a selected gold standard	Results
Kehl et al. (2019) (13)	Lung cancer	Dana Farber Cancer Center	Deep learning NLP models trained on radiological reports to indicate presence of cancer and categorize cancer outcomes as worsening and/or progressing vs improving and/or responding.	Manual abstraction of progression using the PRISSMM framework.	Models reported an AUC of 0.92 for the presence of active cancer, 0.94 for worsening and/or progression, and 0.95 for improving and/or responding.
Kehl et al. (2020) (47)	Lung cancer	Dana Farber Cancer Center	Deep learning NLP models trained on clinical notes to indicate presence of cancer and categorize cancer outcomes as worsening and/or progressing vs improving and/or responding.	Manual abstraction of progression using PRISSMM framework.	Models reported an AUC of 0.94 for the presence of active cancer, 0.86 for worsening and/or progression, and 0.90 for improving and/or responding.
Kehl (2021) (45)	NSCLC and 12 other solid tumors	Dana Farber Cancer Center	Separate deep learning NLP models trained on radiology reports or clinical notes to indicate presence of cancer and categorize cancer outcomes as worsening and/or progressing vs improving and/or responding.	Manual abstraction of disease progression using PRISSMM framework.	Model for NSCLC using radiology reports reported AUCs of 0.97 for any cancer, 0.96 for progression, 0.96 for response, and those trained on clinical notes reported an AUC of 0.95 for any cancer, 0.96 for progression, 0.95 for response.
Arbour et al. (2021) (44)	Lung cancer	Memorial Sloan Kettering	Deep learning NLP trained on radiology reports to determine best overall response with 2 categories (complete response or partial response and stable disease or progression of disease), progression occurring (yes or no) and PFS, with 3 methods of information input used to train the model.	Radiologist assessed RECIST best overall response, occurrence of progression, and date of progression.	The model using input that compared the baseline scan with a mixture of all follow-up scans performed the best. The model reported an AUC of 0.90 for best overall response. Concordance of PFS and RECIST measured progression was and *r*= 0.84.

a AUC = area under the curve; NLP = natural language processing; NSCLC = non–small cell lung cancer; PFS = progression-free survival; PRISSMM = pathology, radiology and/or imaging, signs and/or symptoms, medical oncologist assessment, and biomarkers; RECIST = Response Evaluation Criteria in Solid Tumors

#### Algorithms of changes in treatment

Table 3 lists the studies that used change in treatment to indirectly measure disease progression. The studies reviewed included various definitions of treatment change such as “time to treatment failure” (48-50), “time to next treatment” (11,29,37,43), and “time to treatment discontinuation” (29,39,43,45,51). A treatment may be changed because of failure of a cancer to respond or because of toxicity and/or side effects. However, no studies documented reasons for the treatment change, making it difficu (42) lt to ascertain whether changes were due to cancer progression or treatment side effects. Furthermore, no studies included a comparison of these real-world endpoints to a gold standard for the assessment of the accuracy of their approach. Of note, 3 of the 10 studies examined correlations with overall survival and reported Spearman rank correlation coefficients ranging from ρ equal to 0.36 for “time to next treatment” (43) to ρ equal to 0.81 for “time to treatment discontinuation” (11). However, the expected magnitude of correlation between treatment change or discontinuation and overall survival is unknown and likely to be weaker than the correlation between disease progression and overall survival.

**Table 3.** Example of studies using algorithms of changes in treatment to estimate disease progression in lung cancer patients

Author	Cancer type	Institution	Approach to estimate progression	Comparison to a selected gold standard	Results
Halmos, Tan, Soo et al. (2019) (48)	NSCLC	International study-RealGiDo	Time to treatment failure, defined as time between the first dose of afatinib to the last dose. Time to progression, defined as the time from the first dose of afatinib to the date of first documented progression or death per the patient record.	NA	NA
Reynolds, Masters, Black-Shinn et al. (2018) (49)	NSCLC	McKesson Specialty Health	Time to progression, defined as time between the first dose of crizotinib until date of death due to any cause.	NA	NA
Winfree, Torres, Zhu et al. (2019) (50)	NSCLC	Flatiron Health	Time to progression, defined as the time between the start of IL therapy to the end date of EGFR TKI therapy, date of death, or loss to follow-up.	NA	NA
Stewart, Norden, Dreyer et al. (2019) (43)	NSCLC	US oncology centers	Time to next treatment, defined as the time between initiation of study treatment to the start of a new systemic treatment. Time to treatment discontinuation, defined as time between initiation of aPD-L1 inhibitor to date of discontinuation such as subsequent systemic therapy, gap of more than 120 days after last administration, or death. death ligand	NA^a^	Correlation between overall survival and real-world outcomes, ranged from ρ = 0.36-0.77 for time to next treatment and ρ = 0.46-0.80 for time to treatment discontinuation.
Khozin, Miksad, Adami et al. (2019) (11)	NSCLC	Flatiron Health	Time to next treatment, defined as the time between initiation of a PD-L1 inhibitor to the start of a new line of therapy. Time to treatment discontinuation, defined as the time between initiation of a PD-L1 inhibitor to the date the patient discontinued treatment.	NA^a^	Correlation between overall survival and real-world outcomes (ρ = 0.60 for time to next treatment and ρ = 0.81 for time to treatment discontinuation).
Vilhonen, Kurki, Laitinen et al. (2019) (37)	Lung cancer	Turku University Hospital	Time to next treatment, defined by an automated algorithm using the start of second-line treatment or death as the PFS end date.	NA^a^	Time to next treatment 11.22 months stage III; 7.2 months stage III; real-world PFS 8.5 months stage III; 5.4 months stage IV. Time to next treatment was not a precise estimate of real-world PFS.
Jahanzeb, Lin, Pan et al. (2020) (39)	NSCLC	Flatiron Health	Time to treatment discontinuation, defined as time between initiation of current TKI line to stop: if next line ALK TKI was initiated, if there was a gap of more than 120 days between current therapy and last follow-up date, or death.	NA^a^	Median time to treatment discontinuation (7.04 months) was similar to the median real-world PFS (7.47 months).
Jahanzeb, Lin, Pan et al. (2021) (26)	NSCLC	Flatiron Health	Time to treatment discontinuation; therapy lines for advanced NSCLC were based off of electronic health record documentation of systemic anticancer treatments and were generated by rule-based algorithms indexed to the patient’s diagnosis date.	NA	NA
Bains, Kalsekar, Amiri et al. (2022) (51)	NSCLC	Flatiron Health	Time to treatment discontinuation, defined as the time from index date to therapy discontinuation index date.	NA	NA
Ge, Wu, Jalbert et al. (2022) (29)	NSCLC	Flatiron Health	Time to treatment discontinuation, defined as time from initiation of first-line treatment to second-line therapy or a more than 120-days gap with no receipt of therapy following last treatment dose or death. Time to next treatment, defined as the time between initiation of first-line treatment to second-line therapy or death.	NA	NA

a Study did not use a gold standard, however, they looked at correlations with other cancer endpoints (overall survival, PFS, overall response rate (ORR) and the manually abstracted progression outcome to validate the data. EHR = electronic health records; EGFR= epidermal growth factor receptor; NA = not available; IL= interleukin therapy; NSCLC = non–small cell lung cancer; PD-L1 = programmed death ligand 1; PFS = progression-free survival; TKI= tyrosine kinase inhibitor.

#### Changes in tumor volume

Table 4 lists the approaches used to estimate tumor volumes directly from radiologic images and calculate changes in tumor volume over time as a biomarker of tumor progression (52-54). Although NLP can be used to extract information on tumor progression from the text of the radiology reports, it can be challenging for the algorithm to track tumor changes for a given lesion across text-based radiology reports, especially when more than 1 lesion is present. However, radiologic images contain precise information about the location, size, and appearance of each tumor, making it easier to quantify tumor- or lesion-specific changes. Several studies used a variety of software tools to measure tumor volume and estimate RECIST (55-58). Other studies attempted to correlate volume measurements with pathological response (59) or radiologist segmentation to allow for serial tumor tracking (60).

**Table 4.** Example of studies using tumor volume and measurement approaches to estimate disease progression in lung cancer patients^a^

Author	Cancer type	Institution	Approach to estimate progression	Comparison to a selected gold standard	Results
Oubel, Bonnard, Sueoka-Aragane et al. (2015) (55)	Lung cancer	Median Technologies	Lesion management tool used to compute tumor volume and calculate the RECIST with semiautomated segmentation.	RECIST from RCT	Moderate correlation between RECIST and tumor volume (kappa = 0.553).
Hayes, Pietanza, O’Driscoll et al. (2016) (56)	Lung cancer	Memorial Sloan Kettering	Volumetric measures calculated from CT images using semiautomated segmentation across baseline, first follow-up and second follow-up scans.	RECIST from RCT	RECIST and volume measurements were strongly correlated (ρ = 0.853 at baseline, ρ = 0.861 at first follow-up, and ρ = 0.843 at second follow-up).
Jiang, Hu, Liu et al. (2019) (60)	Lung cancer	Memorial Sloan Kettering	Multiple resolution residually connected network that computes tumor volume using automatic segmentation.	Radiologist segmentation	Dice similarity coefficient of 0.68-0.75 across 3 datasets.
Fusco, Granata, Mazzei et al. (2021) (58)	Lung cancer	National Cancer Institute of Naples	HealthMyne Quantitative imaging decision support tool used to extract tumor volume.	Radiologist measured RECIST	HealthMyne Quantitative imaging decision support tool quantitative volumetric delineation was consistent and matched with radiologist RECIST measurement (80%-84% agreement).
Kidd, Anderson, Cowell et al. (2022) (61)	Mesothelioma	UK Centers	Convolutional Neural Network developed and trained on CT images to determine volumetric response and/or progression following chemotherapy.	Radiologist segmentation and modified RECIST	Human and automated volumes were highly correlated (*r* = 0.851). Human and automated volumes agreed more than half the time (67% of cases; kappa = 0.439). Automated response and modified RECIST agreed in 55% of cases (kappa = 0.24), with higher baseline volume associated with shorter survival time.

a CT = computed tomography; RCT = randomized clinical trials; RECIST = Response Evaluation Criteria in Solid Tumors.

Use of volumetric changes for tracking response is especially of interest for tumors treated with immunotherapy, as prior work has shown that these tumors exhibit changes in both volume and appearance and may exhibit pseudoprogression, which is difficult to capture with standard RECIST measurements (60,61). (. In addition, tumor volumes may also be useful for tumors with complex morphology, such as mesothelioma, which require use of modified RECIST response assessments because of their nonspherical growth (62,63). One study (61) developed an algorithm to automatically segment and measure tumor volume of mesothelioma tumors and reported high correlation (Spearman rank ρ = 0.85) between human-derived volumes and algorithm-derived volumes. By contrast however, another study that used a lesion management tool to compute tumor volume and semiautomated segmentation to calculate RECIST in lung cancer patients reported a moderate correlation (kappa = 0.533) between RECIST and tumor volume (55). The variety of software tools and metrics used to measure tumor volume across the referenced studies may have contributed the heterogeneity in these results. Moreover, there is an underlying need for additional research to identify the validity and feasibility of using tumor volume as a method to measure real-world disease progression.

#### Delta radiomics–based approaches

Table 5 lists studies that attempted to advance image-based approaches beyond tumor volumetric measurements to include other image-based or delta radiomics (64-66) features that may estimate treatment response or be used to track disease progression over time. Only 2 pertinent studies identified in this review used delta radiomics, meaning that the algorithm evaluated changes in features at different imaging time points, usually pre- and posttreatment (67). One study identified a subset of delta radiomic features that correlated with lung tumor size as well as to RECIST response (58). The second study reported the ability to classify response to immunotherapy and to identify pseudoprogression using a subset of the delta radiomics features (68).

**Table 5.** Example of studies using delta radiomics approaches to estimate disease progression in lung cancer patients^a^

Author	Cancer type	Institution	Approach to measure progression	Comparison to a selected gold standard	Results
Fusco, Granata, Mazzei et al. (2021) (58)	Lung cancer	National Cancer Institute of Naples	HealthMyne Quantitative imaging decision support tool used to extract 594 radiomic features including 28 delta radiomics considered to estimate a RECIST-inspired and Choi criteria response.	Radiologist measured volume; RECIST	A total of 13 radiomics metrics were found to be robust and able to quantitatively track reduction or increase in tumor size over time.
Barabino, Rossi, Pampanno et al. (2022) (68)	NSCLC	Italy	Radiomic features (n = 93) extracted from baseline and follow-up computerized tomography images, and the variation, delta, was used to estimate radiological response to immunotherapy. The variation (delta, Δ) was calculated to examine the absolute difference and relative reduction of tumor volume at baseline and follow-up.	RECIST response	A total of 27 features were able to discriminate radiologic response to immunotherapy (*P* values ranging from >.00 to 0.04) and 9 correlated with pseudo progression (*P* values ranging from >.00 to 0.03).

a NSCLC = non–small cell lung cancer; RESIST = Response Evaluation Criteria in Solid Tumors.

## Discussion

Numerous methods have been used to measure or approximate cancer disease progression using real-world data, although no method is without limitations (Table 6). Use of manual abstraction of medical records to ascertain disease progression has advantages, including the full scope of information accessible to the abstractor within the EHR, adaptability to different definitions of progression (RECIST inspired, PRISSMM, etc.), and the flexibility of modifying the abstraction process to meet specific research project criteria. However, there are limitations associated with manual abstraction at scale, such as the length of time required to curate data, the need for highly trained abstractors, and the substantial financial costs to curate large analytic patient cohorts. Additionally, manual abstraction is subject to human error and requires development of strict quality control processes, such as duplicate abstractions, which can increase costs.

**Table 6.** Advantages and disadvantages of the various approaches to estimate disease progression using real-world data^a^

Method	Advantages	Disadvantages
Manual abstraction	Most institutions have easy access to clinical notes or radiology reports within the EHR Existing staff can be trained to abstract data Useful for smaller studies Adaptive to various progression definitions (Response Evaluation Criteria in Solid Tumors and pathology, radiology and/or imaging, signs and/or symptoms, medical oncologist assessment, and biomarkers, etc.) Flexibility of modifying abstraction process to meet specific research needs	Method is not scalable Time consuming and costly Abstractor subjectivity Prone to transcription errors Requires quality checks such as duplicate abstraction; no majorly accepted standard for defining and coding the fields
Treatment changes	Scalable Treatment data can be extracted from structured pharmacy data Treatment data more likely to be fully documented in EHR and/or medical claims Useful method for larger sample studies Faster time to generate research data and at lower cost	Unclear whether treatment changes are because of progression or other causes (eg, toxicity) Algorithms are typically medication or treatment specific and therefore can be difficult to continually update as standard of care therapies for different cancers evolve Results impacted if data are incomplete because of patients receiving part of their care at outside facilities
Natural language processing	Scalable Algorithms can be developed on various EHR text documents Rapidly annotate reports and notes once model is trained Relatively inexpensive once model is developed and trained Can process large volumes of documents Models can adapt as information input and/or format changes overtime	Algorithms require additional training or validation in external datasets Manual abstraction required to train the model Algorithm accuracy may be suboptimal and can decrease with more complex documents Requires expertise of a data scientist to design the algorithms Model accuracy may vary greatly across clinical site and change overtime
Tumor volume	Scalable Output is objective with quantifiable measures of response Use of automatic or semiautomatic segmentation is faster and more accurate than manual segmentation	Requires labeled images for training Quality of segmentation varies by type of method (semiautomatic and automatic) Difficult to choose the segmentation algorithm and match it to the gold standard Difficult to implement in tumors with complex morphology Radiology images may not be readily available retrospectively Different models developed for CT vs positron emission tomography images Methods developed may not be generalizable across institutions Relies only on imaging and therefore doesn’t capture information obtained through other clinical text
Radiomics	Scalable Ability to estimate treatment response using CT scans Access of radiology imaging across institutions using PACS servers	Studies require validation using external datasets Need to define clinical relevance of radiomics measures Response models are built to predict response to specific treatments Impact of heterogenous imaging protocols and need for additional processing Variability in imaging time frame may affect model development Complex process: 1) select and digitize images, 2) selection of area of interest, 3) extract quantitative features, 4) identify biomarkers and train model, and 5) test and validate

a CT = computerized tomography; EHR = electronic health records; PACS = Picture Archiving and Communication System.

Given the widespread prevalence of clinical note dictation, NLP can be a valuable tool for extracting information from clinical notes and/or radiology reports; however, the heterogeneity in types of information documented and the way in which the information is described in the EHR text can hinder the accuracy and the generalizability of NLP. Previous work has also shown that NLP algorithm performance may be dependent on the complexity of the documents processed (47). Initial development of these algorithms may be costly because of the dependency on sophisticated data science expertise. However, once the algorithm is developed, minimal costs are incurred during its use. Therefore, NLP methods may be scalable for the curation of high volumes of clinical notes and/or radiology reports once a model has been developed, trained, and validated.

Of all the methods summarized in this narrative review, algorithms based on a change in treatment are the least complex to define. However, although a change in treatment may signify the cancer failed to respond to the treatment or the occurrence of toxicity and/or side effects, the lack of response may not always indicate progression of the cancer at that point in time. Furthermore, many times the algorithms developed to capture changes in treatment are designed to be treatment and/or medication specific and thus would require continual updates to remain up to date with changes in standard of care. Despite these limitations, a change in treatment is easily documented through medication orders in the EHR and/or insurance claims and can, therefore, be a scalable approach to identifying cohorts of patients for whom the cancer treatment was ultimately unsuccessful in eradicating the disease. We encourage investigators to explore this approach further with these limitations in consideration.

Imaging-based approaches, such as measurement of changes in tumor volume over time, are enticing, given the relatively objective nature of the images themselves. Current disease progression metrics for solid tumors are based on either single (RECIST) (4) or 2-dimensional (World Health Organization(WHO)) (5) imaging-based criteria though volume measurement and have been shown to provide improved tumor assessment (52,56). However, tumor volume measurements are technically challenging to deploy and normally require longitudinally collected image data to infer disease progression. The correlation between tumor volume-based response and RECIST varied across the studies reviewed (55,56,58,59,61), warranting the need for further evaluation of the methods used. In addition to tumor volume, use of delta-radiomic metrics shows promise in improved tumor assessment. Although these more sophisticated imaging-based approaches are theoretically scalable, additional research is needed to determine the clinical value-add of radiomics-based biomarkers for the measurement of disease progression beyond currently known size and volume-based tumor measurements (69). 

Although major advances have been made to develop scalable approaches to estimate disease progression using real-world data, the field continues to be challenged by the lack of a universally accepted gold standard against which to assess the accuracy of each approach. Outside of a clinical trial setting, the alternatives to RECIST for defining real-world PFS used in manual abstraction have been compared with overall survival for the assessment of validity. However, it is unclear what magnitude of correlation should be expected between real-world PFS and overall survival. For example, a systematic review of lung cancer RCTs using anti–programmed death 1 and/or programmed death ligand 1 antibody from 2013 to 2015 reported moderate correlations between PFS and overall survival (ρ = 0.45) (70), which is lower than the magnitude of correlation reported for the association between real-world PFS and overall survival (ρ = 0.61-0.84) (43,45). Therefore, an imperfect correlation between real-world PFS and overall survival is not entirely due to measurement error in the real-world progression estimates.

Similar to RECIST in RCTs, a gold standard in real-world data should be an accurate and widely accepted method for measuring disease progression. In this current study, only 13 of the 40 studies reviewed used a selected gold standard to assess the accuracy among the real-world progression endpoints across their tested methods. However, because there is no widely accepted gold standard in the field of real-world data, the gold standards selected by investigators varied across studies, making it challenging to compare the results on the accuracy of these candidate real-world methods. Although a validated gold standard may not be readily available or feasible to obtain at scale, a method that is not 100% accurate can still be employed to detect the accuracy of real-world disease progression endpoints by serving as a common reference against which other methods could be compared head-to-head. Until a validated gold standard is adopted in this field, we encourage investigators to collectively identify a common reference that can be used to compare other candidate real-world methods in future studies.

Another important factor to consider is the generalizability of these approaches to other cancer types, because of their heterogeneous tumor biology and disease-specific biomarkers used in clinical management. Investigators at Flatiron Health examined whether a real-world progression endpoint, using manual abstraction derived from a cohort of non–small cell lung cancer patients (46), could be applied to 5 additional solid tumor types including metastatic breast cancer, advanced melanoma, small cell lung cancer, metastatic renal cell carcinoma, and advanced gastric and/or esophageal cancer (31). Although the need to incorporate disease-specific information into the definition of real-world progression endpoints for certain cancers (ie, breast, melanoma, small cell lung cancer) was emphasized by the authors, the real-world endpoints constructed for each cancer type positively correlated with overall survival, suggesting that this approach was a generally feasible and accurate approach across various solid tumor types. Moreover, a different study (71) explored the feasibility of expanding their NLP efforts from non–small cell lung cancer patients to 12 solid tumor types including breast, colorectal, endometrial, gastric and/or esophageal, head and neck, leiomyosarcoma, melanoma, high-grade serous ovarian, pancreatic, prostate, renal cell, and urothelial cancer. Interestingly, results from this study suggested that the deep learning models were successful in estimating real-world disease progression with relatively high accuracy, even among cancer types that were not included in model training (71). Nevertheless, there are certain cancers with specific biomarkers that may be indicative of disease progression. For example, circulating tumor biomarker data (72), such as serum prostate–specific antigen testing to determine treatment response in prostate cancer (73) and bone marrow biopsies to assess cytogenic abnormalities in hematologic malignancies (74,75), may contribute to the definition of disease progression by these cancer types. There may be an opportunity to expand approaches for estimating real-world disease progression across multiple cancer types by including circulating tumor biomarker data. Additional efforts are also needed to standardize disease progression definitions across cancer types.

Another possible solution to facilitate use of real-world progression data is to capture the information discretely upstream in the EHR. This would require extensive modifications to EHR systems to include structured fields that capture cancer progression and response in contrast to the existing unstructured clinical notes (76,77). Furthermore, physicians and advanced practice professionals treating cancer patients would require training on how to enter the relevant data using an acceptable standardized definition for cancer progression (76,78,79). Examples of such approaches include the Integrating Clinical Trials and Real-world Endpoints study, which plans to develop methodology to document treatment, disease progression, and toxicity from the EHR with the goal of calculating real-world data endpoints that are comparable to those obtained from RCTs (79). A related initiative is underway at City of Hope in support of the Value-Based Oncology Pathways, including collection of discrete staging data within the EHR with future plans to incorporate disease status of each patient (80,81). Although these initiatives are still nascent and heterogeneous, advances in clinical informatics to transform documentation of cancer progression within the EHR could profoundly enhance the value of real-world data for cancer research.

To our knowledge, this narrative review is the first to provide a broad overview of multiple existing methods to estimate real-world progression in cancer. Although our approach is novel, there are some limitations to note. First, it is possible that the search strategy employed did not identify all pertinent articles that used manual abstraction to estimate real-world progression. For example, some relevant studies previously known to the co-authors that described such approaches (71,82-84) were not included within the 971 articles identified using the search strategy. However, this narrative review did not set out to enumerate the vast number of articles that utilize manual abstraction to measure disease progression as an endpoint for analysis of predictive factors but, instead, presents examples of cancer studies that used distinct and unique methods for the estimation of real-world disease progression, which were difficult to specifically identify in the literature search. Future work reporting on real-world endpoints should be cognizant to tag appropriate Medical Subject Headings terms or keywords to facilitate future review papers.

In summary, there have been major strides in the field of using real-world data to estimate cancer progression at scale. However, additional research is needed to further develop, validate, harmonize, and scale these measures to truly drive discovery in the analysis of real-world data. Consensus on gold standard definitions beyond RECIST is needed to create manually curated datasets for training and validation of automated approaches across centers. Furthermore, head-to-head comparisons of multiple methods against a common gold standard within the same patient populations are needed to gain a better understanding on the relative accuracy of these approaches across different settings. Finally, incorporation of additional disease-specific parameters such as molecular markers (85-87) and patient-reported symptoms (88) may improve the accuracy of algorithms. These future advances in the field can accelerate the use of real-world data in cancer research, creating opportunities for studies of rare cancer subtypes and outcomes research in populations disproportionately underrepresented in cancer clinical trials.

## Supplementary Material

pkad074_Supplementary_Data

## Data Availability

There are no new data associated with this article.
