# Supplementary figures and images for: Overview of approaches to estimate real-world disease progression in lung cancer
Source: JNCI Cancer Spectr. 2023 Sep 21;7(6):pkad074. doi: 10.1093/jncics/pkad074 (PMC10637832; doi:10.1093/jncics/pkad074)

**Supplementary Figure 1.** Selection Criteria for Narrative Literature Review

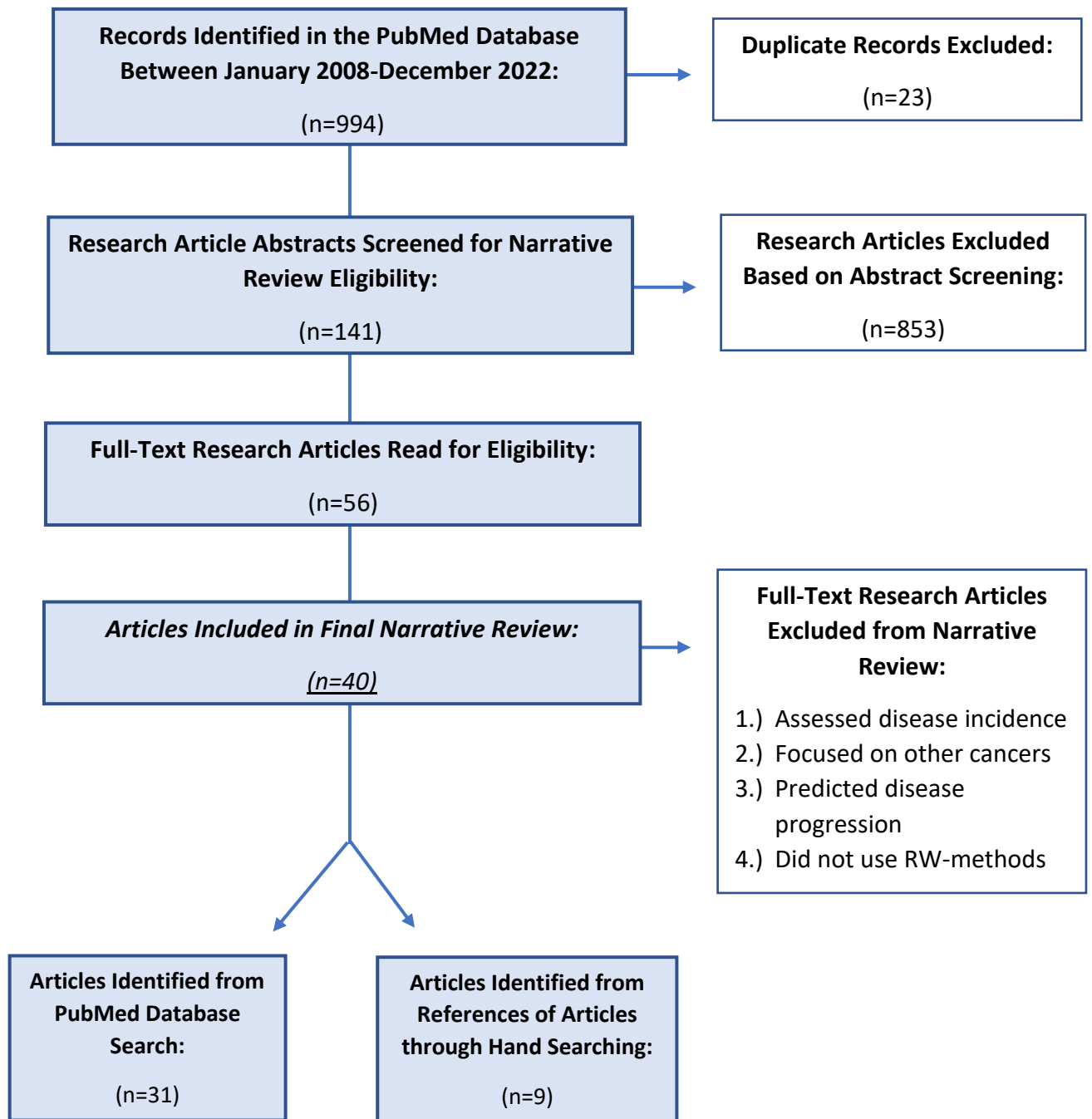

Supplement: pkad074_Supplementary_Data [file pkad074_supplementary_data.pdf]
